# TRAMbio: a flexible python package for graph rigidity analysis of macromolecules

**DOI:** 10.1186/s12859-025-06300-3

**Published:** 2025-10-28

**Authors:** Nicolas Handke, Thomas Gatter, Franziska Reinhardt, Peter F. Stadler

**Affiliations:** 1 Bioinformatics Group, Department of Computer Science & Interdisciplinary Center for Bioinformatics, Leipzig University, Härtelstraße 16–18, D-04107 Leipzig, Germany; 2 Center for Scalable Data Analytics and Artificial Intelligence (ScaDS.AI) Dresden/Leipzig, Leipzig University, Humboldtstraße 25, D-04105 Leipzig, Germany; 3 Max Planck Institute for Mathematics in the Sciences, Inselstraße 22, D-04103 Leipzig, Germany; 4 Department of Theoretical Chemistry, University of Vienna, Währingerstraße 17, A-1090 Wien, Austria; 5 Facultad de Ciencias, Universidad National de Colombia, Bogotá, Colombia; 6 Center for non-coding RNA in Technology and Health, University of Copenhagen, Ridebanevej 9, DK-1870 Frederiksberg, Denmark; 7 Santa Fe Institute, 1399 Hyde Park Rd., 87501 Santa Fe, NM USA

**Keywords:** Rigid graph, Pebble game, (*k l*)-sparse graphs, Protein structure

## Abstract

**Background:**

Insight into the rigidity or flexibility of molecular structures is integral for a series of common research questions in molecular biology, including the identification of functional regions, simulated protein unfolding, or tracking and prediction of conformational changes in proteins over time. Determining rigidity in 3-dimensional space is a difficult problem in general. For a well-defined subclass of frameworks, however, this task can be solved in polynomial time with the help of constraint counting algorithms known as pebble games. Although this approach is well established, no easy-to-use implementation of the pebble game algorithm in the context of general graph analysis and molecular rigidity is currently available to researchers.

**Results:**

To close this gap, we developed TRAMbio, a Python-based software tool for Topological Rigidity Analysis in Molecular Biology. We summarize and discuss the theoretical foundation of the pebble game and how it can be applied to molecular rigidity.

**Conclusions:**

TRAMbio performs well even on large molecules and on discrete time series of protein movement. Results are accessible for both bioinformaticians and biologists, as rigid components can be rendered using standard molecular visualization platforms.

## Introduction

Proteins constitute the majority of a cell’s dry mass and govern the key processes of most cellular functions [1]. They catalyze most chemical reactions of the cell’s metabolism, act as regulators for the production and degradation of biological macromolecules, exert control in response to environmental cues, and orchestrate development in multicellular organisms. As a consequence, alterations of protein structures caused by mutations, chemical modification, or triggered by pathogens such as bacteria or viruses are often the molecular root cause of diseases through changes of binding interfaces and changes of interactions that disrupt regulatory network.

Since physical and chemical interactions with their environment define protein function, accurate representations of their three-dimensional structure are a critical first step towards understanding their biological roles. A wide variety of experimental techniques has been developed to determine single 3D structures of proteins: most commonly, nuclear magnetic resonance (NMR), cryogenic electron microscopy (cryo-EM) and X-ray crystallography that provide a time invariant structure are employed. Recently, dramatic progress has also been achieved with regards to the computational prediction of protein structures, at least as far as naturally evolved protein sequences are concerned [2–4].

Conformational changes, are an important part of the mechanism underlying protein function. Dynamic changes of protein subsystems, however, are much harder to capture experimentally than a single, dominant structure. Although NMR can in principle contribute to measuring protein dynamics [5], computational approaches are in general more accessible and widespread. Most commonly Molecular Dynamics (MD) is employed to simulate the motions of individual atoms based on potentials describing chemical bonds as well as electrostatic and other interactions within and between molecules.

A downside of MD simulations is that the atom-level resolution makes it non-trivial to identify the mechanics behind non-local changes of the structure. This is of particular relevance in allostery, where a binding event at one site affects a different functional site [6, 7]. In such applications it is of particular interest to identify substructures that move collectively relative to each other.

Rigidity analysis, reviewed in [8, 9], is a promising computational strategy to identify such blocks. To this end, interatomic distances that remain essentially unchanged over larger time-scales are interpreted as edges of a graph. Rigid components that do not admit internal motions but retain residual degrees of freedom relative to each are then defined at a purely graph-theoretical level. The general usefulness of rigidity analysis in protein science has been demonstrated e.g. in [10–14].

Efficient combinatorial algorithms [15, 16] are based on so-called pebble games [17]. Although practical implementations, e.g. ProFlex/FIRST [10], ASU FIRST [18] and KINARI [19–21], have become available, an open-access tool for the analysis of molecular rigidity does not seem to be available at present. Here we aim to close this gap. TRAMbio implements the classic pebble game algorithms for rigidity and at the same time provides a convenient framework geared towards protein analysis, including a direct interface to the Protein Data Bank. Despite the focus on protein structures, TRAMbio implements at its core a general class of pebble game algorithms that handle so-called $(k,\ell )$-sparse graphs, which encompass the most important notions of graph rigidity. TRAMbio thus can be used to solve combinatorial rigidity problems independent of a specific application domain.

This contribution is organized as follows. The theoretical background on graph rigidity together with the pebble game algorithm and its application to molecular structures are briefly summarized in Sect. 2. The software tool TRAMbio is presented in Sect. 3, detailing its architecture and functionalities as well as insights on implementation decisions. Section 4 provides a showcase example and an evaluation of the required computational resources. An outlook to open issues and future developments is the main topic of the concluding Sect. 5.

## Theory

### Rigidity and (*k*, *l*)-sparse graphs

**Fig. 1:**
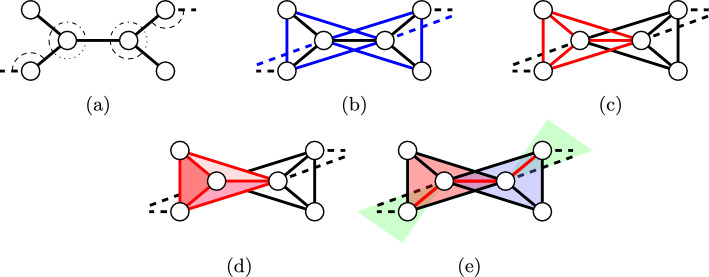
A molecular bar-and-joint framework with fixed bond-angles (**a**) and the corresponding bond-bending framework (**b**). Each vertex with its covalent neighbors forms a complete subgraph (**c**) which corresponds to a rigid body (**d**). Every two adjacent bodies share a single edge (red lines in (**e**)) which only permits a hinge rotation around the bond axis. Figure adapted from [23]

Molecules in general have well-defined bond lengths and bond angles (cf. Fig. 1a). The latter imply that also next-nearest neighbors have fixed, well-defined distances. Starting from a molecular graph $M=(V,B)$, where vertices represent atoms and edges the chemical bonds, the fixed distances are encoded in a second graph $G=(V,E)$ with $xy\in E$ whenever there is $z\in V$ such that both $xz\in B$ and $zy\in B$ (cf. Fig. 1b). Accordingly, this graph describing all rigid distances *G* on the set of atoms *V* is the so-called square of *M* [22]. The atoms *V* are embedded in *D*-dimensional Euclidean space by a map $\eta : V\rightarrow \textbf{R}^{D}$, where $D=3$ for molecules. The graph *G* together with the map $\eta$ and the length function $\ell (xy):=\Vert \eta (x)-\eta (y)\Vert$ for all $xy\in E$ is called a *framework*. An Euclidean distance matrix $\textbf{D}_{xy}:=\Vert \eta (x)-\eta (y)\Vert$ for all $x,y\in V$ is naturally associated with the framework such that $\textbf{D}_{xy}=\ell (xy)$ for all $xy\in E$. A framework $(G,\eta )$ is globally rigid if its distance matrix $\textbf{D}$ is uniquely defined by the edge lengths $\ell$.

An infinitesimal motion $\textbf{v}$ consists of a *D*-dimensional velocity vector $\textbf{v}_x$ for each $x\in V$ such that $(\textbf{v}_x-\textbf{v}_y)(\eta (x)-\eta (y))=0$ for all $xy\in E$. This linear system can be rewritten in the form $\textbf{R}_{\eta }\textbf{v}=0$, where $\textbf{v}$ is a *D*|*V*|-dimensional vector of velocities and $\textbf{R}_{\eta }$ is the $|E|\times D|V|$-dimensional *rigidity matrix*. Each row of $\textbf{R}_{\eta }$ corresponds to an edge *xy* of the graph *G*, with entries $\textbf{v}_x-\textbf{v}_y$ for the velocity of vertex *x*, $\textbf{v}_y-\textbf{v}_x$ for the velocity of vertex *y*, and 0 otherwise. For an example we refer to [24].

The framework $(G,\eta )$ is infinitesimally rigid if there are only “trivial” motions $\textbf{v}$ that do not affect the distances between any two vertices, i.e., if the rank of $\textbf{R}_{\eta }$ equals $D|V|-{D+1 \atopwithdelims ()2}$. The ${D+1 \atopwithdelims ()2}$ trivial degrees of freedom correspond to the translation, rotation, and general screw motions of a rigid body in *D*-dimensional space. The rank of $\textbf{R}_{\eta }$ in general depends explicitly on $\eta$. However, if a graph *G* has an infinitesimally rigid framework $(G,\eta )$, then *all* its generic frameworks (i.e., those with embedding coordinates algebraically independent over the rational numbers) are infinitesimally rigid [25]. Generic infinitesimally rigidity, therefore, is a property of the graph *G* only.

For $D=2$ dimensions, generic rigidity of bar-and-joint frameworks is characterized by the Geiringer-Laman theorem [26, 27]: $G=(V,E)$ is generically rigid as a bar-and-joint framework in 2 dimensions if and only if it contains a spanning subgraph $G'=(V,E')$ with $|E'|=2|V|-3$ and edges for all subsets of edges $F\subseteq E'$ holds $|F|\le 2|V(F)|-3$, where *V*(*F*) is the set of vertices incident with *F*. An analogous counting formula for $D=3$ (with $|E'|=3|V|-6$ and $|F|\le 3|V(F)|-6$ according to Maxwell), however, fails due to the famous “double banana” graph [28].

As noted above, molecules are naturally modeled as bar-and-joint frameworks of the square of the structural formula. Alternatively, each atom and its neighbors can be viewed as rigid bodies connected by hinges corresponding to the connecting bonds, which in general allow free rotations, cf. Fig. 1d and e. However, these molecular body-and-hinge frameworks have a special configuration called hinge-concurrent, because all the hinges (chemical bonds) incident to a body intersect at the atom at the center of the body. This special configuration in principle could change the (infinitesimal) degrees of freedom by introducing additional linear dependencies. Rigidity of 3D hinge-concurrent body-and-hinge frameworks is equivalent to rigidity of corresponding rigid panel-and-hinge frameworks [29]. In 1984, Tay and Whiteley [30] conjectured that, in arbitrary dimensions, a multigraph *G* can be realized as an infinitesimally rigid body-and-hinge framework if and only if *G* can be realized by an infinitesimally rigid panel-and-hinge framework. This so-called “Molecular Conjecture” was proven by Jackson and Jordán for 2 dimensions [22] and finally by Katoh and Tanigawa for arbitrary dimensions [31]. In particular, therefore, it suffices to test body-and-hinge rigidity of a molecular graph to determine bar-and-joint rigidity of the square of the molecular graph *G* and thus rigidity of the molecule.

**Table 1:** Rigidity problems equivalent to $(k,\ell )$-tightness

Framework type	*k*	$\ell$	References
2D bar-and-joint	3	2	[27]
2D body-and-bar	3	3	[30]
*Frictional jamming*	3	3	[32]
*Translational rigidity*	3	4	[33]
3D minimally rigid	3	6	[34]
3D body-and-bar	6	6	[30]

A framework is minimally rigid if it is rigid and the deletion of any edge leads to a flexible (i.e., non-rigid) framework. Several different types of generic rigidity of graphs are characterized by counts of degrees of freedom, which leads to combinatorial conditions analogous to 2-dimensional bar-and-joint frameworks, see Table 1 for some important rigidity models. The mathematical basis is conveniently expressed in terms of so-called $(k,\ell )$-sparse graphs, where $k>0$ and $0\le \ell <2k$. This class of graphs was introduced in the context matroid theory [35]:

#### Definition 1

A multigraph $G=(V,E)$ is $(k,\ell )$*-sparse* if $|E'|\le k|V(E')|-\ell$ for all $\emptyset \ne E'\subseteq E$. It is $(k,\ell )$*-tight* if it is $(k,\ell )$-sparse and $|E|=k|V|-\ell$.

The $(k,\ell )$*-tight* graphs thus correspond to minimally rigid graphs for the corresponding notion of rigidity. That is, a graph is “$(k,\ell )$-rigid”, if it contains a spanning $(k,\ell )$-tight subgraph, i.e., it is $(k,\ell )$-sparse.

#### Definition 2

A $(k,\ell )$*-rigid component* is vertex-maximal subgraph of *G* that is $(k,\ell )$-tight.

Two $(k,\ell )$-rigid components are always edge-disjoint. They are vertex-disjoint for $0<\ell \le k$ and may overlap in at most one vertex for $k<\ell <2k$. For $\ell >0$, all $(k,\ell )$-rigid components are connected subgraphs [16]. For the special case of $\ell =0$, only a single $(k,\ell )$-rigid component exists in a given graph.

### Pebble game algorithms

**Fig. 2:**
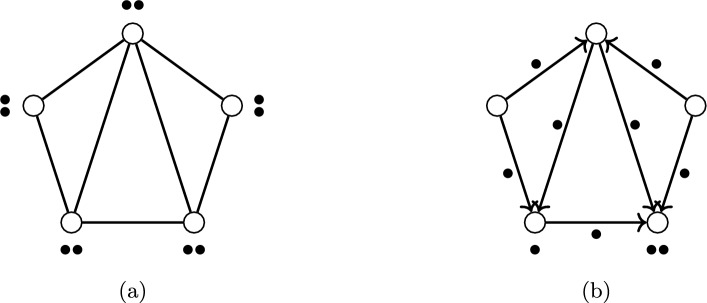
A graph *G* with 2 pebbles assigned to each vertex (**a**), for which it is possible to cover every edge with a pebble (**b**). Each edge is directed away from the node where the covering pebble was used from. Therefore, in every generic embedding of *G* in the plane, all edges are independent

**Fig. 3:**
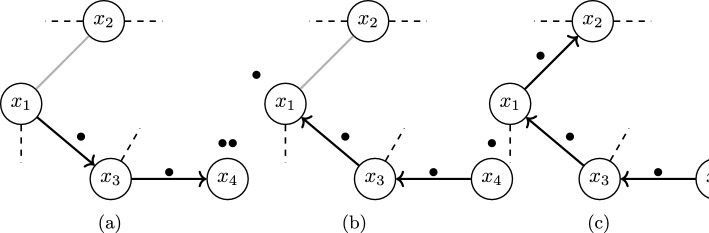
A graph during the pebble game. Inserting the edge $x_1x_2$ (gray) requires freeing a pebble (**a**). A pebble from $x_4$ can be used to cover the edge $x_3x_4$, which frees a pebble on $x_3$ to cover $x_1x_3$, finally freeing a pebble on $x_1$ (**b**) that can cover the new edge $x_1x_2$ (**c**)

A pebble game is a single-person game played on a set *V* of nodes each of which is initialized by *k* pebbles. The player inserts and orients edges between the vertices. The rules of a particular pebble game determine conditions under which an edge is accepted, when pebbles can be moved, and edges are re-oriented (cf. Fig. 2). A pebble game algorithm “plays” the pebble game by attempting to insert the edges of a given graph *G* in arbitrary order. Jacobs and Hendrickson proposed a simple pebble game algorithm to decide planar bar-and-joint rigidity, i.e., to recognize Laman graphs [17]. This idea was generalized in [16] to $(k,\ell )$-graphs:

Starting from an edge-less graph *D*, an edge *e* of the input graph *G* is inserted into *D* between two nodes *u* and *v* if the two vertices together contain at least $\ell +1$ pebbles. Moreover, no more than *k* pebbles are allowed at any single vertex. Otherwise additional pebbles can be retrieved consecutively from any vertex *w* that is reachable from *u* or *v*. Transporting a pebble from *w* to *u* or *v* inverts the orientation of all edges along a single directed path from *u* to *w* or *v* to *w*, respectively. The edge is directed $u\rightarrow v$ and the pebble in *u* is removed, unless no pebbles are present on *u* in which case the roles of *u* and *v* are reversed (cf. Fig. 3). If *D* contains exactly $\ell$ pebbles after attempting to insert all input edges, then *G* is $(k,\ell )$-tight if all edges were inserted successfully, otherwise it contains redundant edges.

Rigid components can be detected by a simple extension of this procedure that explicitly maintains the rigid components. Upon attempting to insert an edge $e=uv$ one checks if *u* and *v* are in the same component, in which case the edge is redundant. Otherwise, if *e* can be inserted, one checks if a new component is formed or if the additional edge causes two or more components to join.

### Representation of molecular structures

For molecular structures, the rigidity problem can therefore be solved by the (6, 6)-pebble game on the multigraph which underlies the molecular body-and-hinge framework. Every molecular single bond is encoded as 5 edges between the same pair of atoms in the body-and-hinge graph, leaving a single degree of freedom given by the torsion angle. Double bonds, on the other hand, are rigid and constrain the neighborhoods of the incident atoms to a common plane. This additional constraint is represented as a 6-th edge in the multigraph. Triple bonds are treated analogously.

**Fig. 4:**
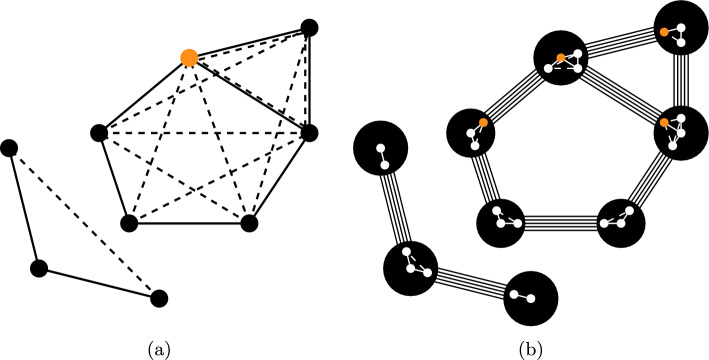
A bond-bending framework in 3-space (**a**) and the multi-graph of the corresponding body-and-bar framework (**b**). The dashed lines represent the next-nearest neighbor edges. The inside of each body in (**b**) shows the set of vertices from (**a**) that consolidate this body. The orange dot marks the same vertex from the bond-bending framework. Figure adapted from [36]

Since the result of the pebble game is the list of $(k,\ell )$-tight subgraphs in this “molecular multigraph”, where each node represents a rigid body consisting of a central atom with its nearest neighbors, these results need to be mapped back onto the molecular graph. An atom may be part of more than one rigid component, since it may be both at the center of a rigid body and at the periphery of one or more rigid bodies centered at each of its adjacent atoms (cf. Fig. 4b). Any atom of valency 1, e.g., hydrogens and halogens, could also be considered to be forming a rigid body together with their unique binding partner. These are completely subsumed in the adjacent body, however, and hence can be ignored. In Fig. 4, the three vertices on the bottom-left are already represented by a single rigid body.

In applications to protein structures, interesting results can be expected only if hydrogen bonds and other contacts are taken into account. Otherwise, at least the torsion angles between N-C$_{\alpha }$ and C$_{\alpha }$-C along the backbone [37] remain unconstrained^1^ and there are no “interesting” rigid components beyond parts of individual amino-acids. Hydrogen bonds are naturally treated like singe covalent bonds due to their well-defined bond lengths and bond angles.

The representation of additional, weaker non-covalent interactions in rigidity analysis is much less straight-forward. This concerns in particular hydrophobic tethers as well as weak hydrogen bonds. One possibility is to represent such contacts with fewer than five edges, advocated in particular in [9, 18, 38, 39]. In generic body-and-bar frameworks this would directly relate to fewer constraints between the respective bodies and thus a representation of more flexibility in the overall system. With respect to molecular modeling, however, there may appear further linear dependencies through the intersection of chemical bonds incident to a body. In addition to the previously mentioned hinge-concurrencies, this model would also allow for the intersection of two bars or a bar and a hinge, termed bar-bar- and bar-hinge-concurrencies, respectively [40]. While the “Molecular Conjecture” implies that hinge-concurrency does not affect the rigidity results, no analogous results exist for bar-bar- or bar-hinge-concurrencies. Moreover, the use of specific edges to represent such contacts should be regarded as a heuristic approximation [41].

## The TRAMbio package

TRAMbio (Topological Rigidity Analysis in Molecular Biology) is a pure-Python package based on and centered on the pebble game. It provides functionality for general applications in graph theory including testing for $(k,\ell )$-sparsity as well as determining the $(k,\ell )$-spanning subgraphs, i.e., the $(k,\ell )$-rigid components. With regards to molecular data, in particular proteins, TRAMbio provides tools for the rigidity analysis on an atom or residue-level with further functionality towards specialized tasks like (a) simulated protein unfolding [11] on single-state protein data and (b) time-based tracking of rigid component changes in molecular dynamics (MD) trajectories.

### Input data

TRAMbio processes general graph data in GraphML^2^ format and provides convenient interfaces to PDB and XTC data to study rigidity in protein structures.

**Fig. 5:**
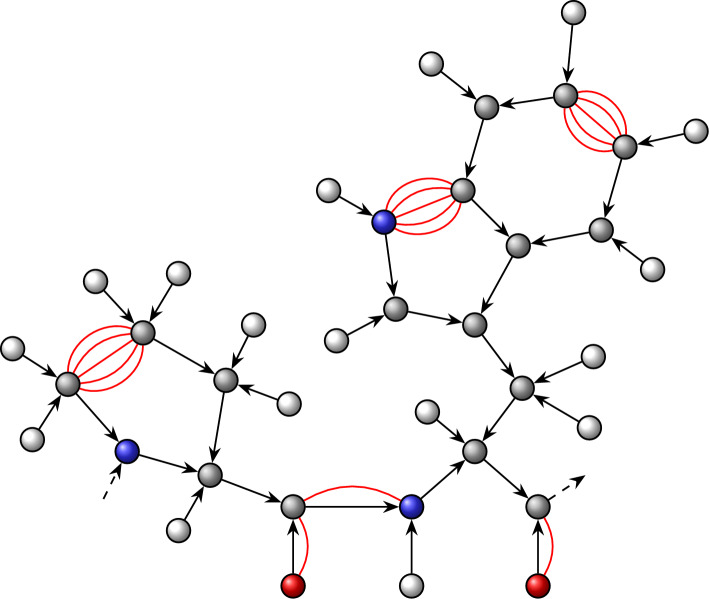
Example of the pre-constructed pebbled graph for a protein chain with amino acids proline (left) and tryptophan (right). Every directed edge represents five edges in the pebbled graph. The red edges are later added through the actual pebble game. Figure based on images from [44]

The PDB file format is a text-based format introduced in 1972 to store three dimensional protein structures in the Protein Data Bank, the main community resource for this type of data [42]. At the time of writing, the PDB provides 229, 681 high quality structures for proteins, with more computational results. PDB data record atom coordinates. Covalent bonds, hydrogen bonds, and other interactions are not given explicitly and thus need to be inferred from relative positions of atoms. TRAMbio implements algorithms for extracting the graph structure following well-established procedures: (a) Covalent interactions are determined by firstly using amino-acid templates (cf. Fig. 5), as in the initial step of KINARI’s “phased pebble game” [21], and then by employing a general distance-based algorithm for missed edges that is essentially identical to the method used in graphein [43]. (b) Hydrogen bonds and salt-bridges are determined as described in [11]. (c) Hydrophobic interactions are calculated using either the distance-based approach based on Van der Waals surfaces [11] or the potential-based algorithm described for KINARI [40] leveraging a Lennard–Jones-12-6 potential. (d) The remaining non-covalent interactions (e.g. pi-cation or pi-stacking) are calculated using the geometric parameters listed at https://getcontacts.github.io/interactions.html. Further details on the construction process for molecular graphs can be found in Appendix A.

Molecular dynamic trajectories describe the temporal evolution of the three-dimensional conformations of a molecule. The XTC file format encodes the trajectories as time series of 3-dimensional coordinates for atoms, based on the unix concept of External Data Representation. After loading the trajectory with either MDAnalysis [45, 46] or mdtraj [47], the coordinates of each selected frame are exported as PDB data and the interactions between atoms are calculated as mentioned above. Since all frames model the same protein in various states, it suffices to determine covalent bonds once and then assumed for all frames while the non-covalent interactions are calculated for each frame individually.

### Tool description and architecture

The architecture of TRAMbioconsists of two main parts. At its core lies an API for the general $(k,\ell )$-component pebble game on arbitrary *k* and $\ell$ as well as the specific (6, 6)-pebble game in the context of molecular rigidity calculation.

Workflow structures follow the SOA (service-oriented architecture) design pattern, implementing convenient interfaces for the pebble game API. Although most commonly associated with Web-Applications, SOA serves as a useful design foundation for software systems consisting of distributed yet integrated components. In the case of TRAMbio, aside from the capability of directly downloading protein information from the Protein Data Bank,^3^ the remainder of the tool and especially the entire calculation runs completely locally. The usage of SOA separates program logic of TRAMbiointo dedicated registries for handling I/O, internal structures, molecular interaction modeling, and the overall workflows. Although Python does not have a native interface structure, the usage of abstract classes allowed for a recreation of interface functionality.

The internal data structures and the implementation of the pebble game highly leverages the Python packages NetworkX [48] and BioPandas [49]. Input data of proteins is stored and processed in PDB format through the use of data frames. Aside from that, the most notable internal structures are (1) a graph representation of the protein at atom level, with detected covalent and non-covalent bonds as annotated edges, and (2) a weighted bi-directional digraph serving as an auxiliary graph during the pebble game. Weighted digraphs are utilized in contrast to multigraphs based on an overall performance increase, as edge inversions (removal and re-adding of individual edges) are reduced to in practice more time efficient^4^ weight shifts. This concept was already applied in KINARI-2 [21].

A customized file format for storing molecular rigidity or general pebble game results was designed based on XML (eXtensible Markup Language). XML-Schema are provided for both structure types to warrant file integrity. Since the pebble game employs an all-atom model for rigidity calculation, additional functionality is provided to convert the results to residue-level rigid components. Due to its reduced complexity, these outputs are formatted in JSON (JavaScript Object Notation).

### Functionality, features, and implementation

This subsection provides an overview of TRAMbio’s workflows in general and specifically geared towards application on single state proteins and trajectories from molecular dynamics (MD) simulations. It also highlights key features and underlying implementation details. TRAMbio in particular reimplements^5^ some functionalities of ASU FIRST [10, 18] and KINARI [19, 21] in an open-source framework that is designed to be easy to maintain and extend.

Aside from the mentioned service-oriented API, the TRAMbio is a command-line-only tool featuring five separate workflows (see Table 2).

**Table 2:** Command-line workflows provided by TRAMbio

Nr.	Command	Description
1.	tram-pebble	Applies the $(k,\ell )$-pebble game on general graph data
2.	tram-pdb	Calculates rigid components and dilution analysis for single state PDB-data
3.	tram-xtc	Calculates rigid components in protein trajectories
4.	tram-residue	Converts the atom-level results of 2. and 3. to residue-level components
5.	tram-pymol	Generates source files for result visualization with PyMol [50]

Leveraging the known covalent structure of biopolymers such as DNA, RNA, and in this case proteins, the majority of edge insertions corresponding to covalent bonds can already be decided prior to running the pebble game. Thereby, a reverse arborescence,^6^ with each node having at most $\ell -1$ edges pointing away, is extracted from the protein graph. This represents a well-defined state of the pebble game. This approach, first introduced as “phased pebble game” by Bygi and Streinu [21], greatly reduces the computation time required, since only the remaining edges need to be tested through actual runs of the pebble game. To further speed up computation, a multiprocessing approach is used for state-wise pebble game runs on trajectories and for mapping results to rigid molecular structures.

One major design consequence of building a pure-Python package relates to the mechanical modeling of molecules, especially the calculation of non-covalent interactions. This excluded the use of third-party software for out-sourcing model complexity. The off-the-shelf solution of the existing Python package graphein [43] for molecular graph modeling was temporarily considered during development. However, we realized that its purely distance-based interaction model is too limited. The current version of graphein (v. 1.7.7 when this study was conducted) determines hydrogen bonds as follows: first, all atoms of the molecule are filtered into a set of donor and acceptor atoms using a set of pre-determined rules for each residue-atom-type. Then, all pairs of atoms (not limited to just donor-acceptor pairs) in that set are considered to be connected by a hydrogen bond, if they are at most 3.5, ${\stackrel{\circ}{\text{A}}}$ apart (or 4.0, ${\stackrel{\circ}{\text{A}}}$, if the pair contains a sulfur atom). In contrast, ASU FIRST [11] uses geometric criteria and a Mayo-potential [51] to model of hydrogen bonds. Since this approach appears to be better suited for our requirements we implemented this interaction model directly within TRAMbio. This decision coupled with the SOA structure pattern provides high flexibility, as each interaction is isolated through its own parameterized service and it is possible to expand the molecular interaction model by introducing additional services.

Protein unfolding can be understood in terms of breaking weak interaction in response to increasing temperature. The matroid nature of $(k,\ell )$-sparse graphs ensures that the insertion order of edges is arbitrary. Thus, it suffices to sort non-covalent bonds from strongest to weakest bond and to record rigid components at each step of this insertion order. Protein unfolding, accordingly, can be simulated in a single run of the pebble game, considering the dissolution of rigid components in reverse insertion order [21].

As mentioned before, the current version of TRAMbio is a command-line-only tool and does not provide its own graphical visualization functions. Instead, it features a separate workflow for creating annotated, PyMol-compatible files. The application examples shown below have been generated in this manner. TRAMbio utilizes a consistent-coloring algorithm, similar to the one proposed in [52], to allow a visual tracking of rigid structure development over time (for trajectories) or during dilution (for individual states).

## Applications

In this section we summarize TRAMbio’s runtime performance on general graphs as well as selected proteins, and explain the visualization of result on an exemplary dilution analysis.

For benchmarking on general graphs, randomized Laman graphs and random graphs following the classic Erdős–Rényi model with 2 distinct edge probabilities were generated, as well as a set of complete graphs, with varying node sizes. TRAMbio was run using tram-pebble mode. Results are summarized in Table 3.

For small graphs, runtimes are dominated by computational overhead such as IO-operations rather than the pebble game algorithm. For larger graphs, the overall runtime appears to scale close to linearly in the number of edges. While the pebble game itself has a runtime complexity of $O(n^2)$, it is easy to observe that the pebble game can be played in *O*(*n*) on path graphs. For arbitrary graphs we therefore also gain a factor *n* on a path acting as a ”backbone“ in each graph component. The second factor of *n* derives from structures branching of this path. However, we generally expect there to be only a number $m<<n$ of such structures, leading to the observed behavior.

**Table 3:** Overview of the runtime for tram-pebble on various graphs for $k=2$ and $\ell =3$

Graph type	Nodes	Edges	Single-core		Multi-core	
			Real time	CPU-Time	Real time	CPU-Time
			(mm:ss.ms)	(mm:ss.ms)	(mm:ss.ms)	(mm:ss.ms)
Laman	10	17	00:01.16	00:00.89	00:01.45	00:00.92
	100	197	00:01.22	00:00.95	00:01.31	00:01.04
	1000	1997	00:10.92	00:10.60	00:07.26	00:30.14
Random (0.3)	10	13.7	00:01.51	00:00.93	00:01.17	00:00.93
	100	1485.9	00:01.86	00:01.24	00:01.47	00:01.20
	1000	149,850	01:48.56	01:47.83	00:56.34	01:05.42
Random (0.5)	10	21.5	00:01.22	00:00.81	00:01.35	00:01.04
	100	2487.3	00:01.57	00:01.26	00:01.71	00:01.40
	1000	249,750	03:11.13	03:10.30	01:28.30	01:32.64
Complete	10	45	00:01.31	00:00.99	00:01.16	00:00.93
	100	4950	00:01.96	00:01.60	00:01.82	00:01.61
	1000	499,500	06:37.01	06:35.62	02:44.84	02:56.68

Calculations were run on a system with Intel® Xeon® Gold 6130 using either one (Single-Core) or up to 60 cores (Multi-Core). The exact number of cores used for parallelization depended on the respective graph decomposition using a Greedy-type algorithm. The parenthesized value for random graphs denotes the edge probability. Since the Laman and Random graphs are not uniquely determined, the reported times are averages over runs of 10 different graphs each. For these graph types, we report the average number of edges over a sample of 10 graphs

Although TRAMbio already features a variant of the pebble game which uses parallelization, all graphs in Table 3 were analyzed once using single-core processing for an easier comparison of runtimes. Since the order of inserted edges has no influence on the detected components, it is theoretically possible to separate a graph into disjointed sub-graphs and a set of joining edges, run pebble games (in parallel) on these sub-graphs, combine their resulting pebbled graphs, and finally play the pebble game for the joining edges. Due to the already near-linear performance of the pebble game [16, 17], the computational overhead related to splitting and re-combining the respective graphs appears to be prohibitive on smaller graphs, resulting in almost identical real-time performance. However, for a sufficiently large number of edges, multiprocessing can significantly decrease computation times. Use cases and size thresholds for efficient multiprocessing for general case graphs are yet to be determined. As of date, these features are still considered experimental.

Benchmarks on real datasets were performed on publicly available protein data, utilizing two different modes of TRAMbio. Rigidity analysis was performed for six different protein PDBs with tram-pdb and trajectory-analysis with tram-xtc on a single protein-interaction in two different time resolutions. Runtimes are summarized in Table 4. PDB data was downloaded from the Protein Data Bank prior^7^ to execution and missing hydrogen atoms were added using the software Reduce [53]. Benchmarks do not include times for both. Parallelization was permitted for trajectory-analysis, as frames can be computed largely independent of each other. The trajectory data of the $\beta _2$-adrenergic receptor [54] was analyzed once with a stride of 50 (only using every 50-th frame) and once on the entire trajectory. All runs were performed using the default run options, which mainly includes the identification of all possible non-covalent interactions, as described in Sect. 3.1.

**Table 4:** Overview of the runtime for the analysis of single-state PDB data (upper section) and trajectory data (lower section)

Protein name	# Atoms	Max RAM	Real time	CPU-Time
		(in GB)	(HH:mm:ss.ms)	(HH:mm:ss.ms)
Trp-Cage Miniprotein Construct TC5b (1L2Y)	304	5.8	00:00:01.45	00:00:01.21
Titin module A71 from Human Cardiac muscle (1BPV)	1603	5.8	00:00:02.86	00:00:02.60
Bovine rhodopsin (1L9H)$^{*}$	6039	6.9 (5.7)	00:00:13.26	00:00:10.02
Bovine bile-salt activated lipase complexed with taurocholate (1AQL)$^{*}$	16,798	15.6 (5.9)	00:01:00.09	00:00:56.55
Native human TSH bound to human Thyrotropin receptor in complex with miniGs399 (7T9I)	23,613	25.3 (6.1)	00:04:32.39	00:04:30.92
Recombinant thiocyanate hydrolase (2ZZD)$^{*}$	34,776	48.2 (7.2)	00:09:18.80	00:09:14.18
Copper amine oxidase from *hansenula polymorpha* (1A2V)$^{*}$	63,926	149.5 (9.8)	00:55:33.35	00:55:23.30
$\beta 2$-adrenergic receptor without G protein [54]	4574·61 frames	$6.8 + 3^{*}$	00:01:28.94	00:13:03.68
	4574·3001 frames	$7.2 + 5^{*}$	00:23:34.97	03:56:54.99
$\beta 2$-adrenergic receptor with coupled GDP-bound Gs protein [54]	16,646·63 frames	$15.4 + 6^{*}$	00:02:50.27	00:38:07.03
	16,646·3132 frames	$15.4 + 10^{*}$	01:32:31.00	19:30:08.95

Calculations were performed on a system with Inte®l Xeon® Gold 6130 using up to 64 CPUs with default CLI options. For the proteins marked with an asterisk ($^*$), no hydrogen atoms were present in the PDB records and their positions were approximated via Reduce [53]. The max RAM column reports the maximum amount of RAM allocated at once during the computation (rounded up). Parenthesized values additionally report the maximum amount of allocated RAM after the initial step of loading input data. Since trajectories are calculated via multiprocessing, the reported RAM value is only for the master process with the approximated summed value for simultaneous sub-processes added on top. The latter one is marked with an asterisk ($^*$). The available RAM for each run was limited to 200 GB

Compared to general graphs, on the same number of atoms/nodes, runtimes are on average longer by a significant factor. Concurrently, runtimes delineate more strongly from linear complexity, showing a higher influence of the theoretical quadratic term of the pebble game. We presume both effects are due to the extension of the classic pebble game algorithm for tram-pdb by dilution analysis. Dilution analysis constitutes an integral step for protein analysis, as will become apparent shortly, offering valuable data for post-processing. It is therefore deeply integrated into the algorithm, creating a snapshot for the interpretation of the state of the pebble game each time the components change from an inserted hydrogen bond (or other quantified non-covalent interactions). Runtime costs for this additional step largely exceeding those of the original pebble game. In contrast, TRAMbio mode tram-xtc for trajectory analysis does not include dilution analysis, resulting in running times of around 22-36 s for single frames with 16,646 atoms (cf. Table 4) which is considerably faster than a dilution analysis on a comparatively size PDB.

Initially, it was considered to treat the dilution analysis as an optional feature and by default to run the rigidity analysis without dilution. However, this led to significant problems in the user experience of researchers. In a typical setup, the user has to provide an energy *threshold*, up to which bond strength hydrogen bonds should be considered, thereby defining the set of relevant bonds, heavily influencing rigid components. Given the energy function [11] used to model the strength of hydrogen bonds in TRAMbio, however, it is difficult to derive exact local bond energies (or a corresponding system temperature for that matter). Accordingly, the set of actually inserted hydrogen bonds may differ from user expectation. Adaptions to the threshold are trivial to determine if dilution data is available, but require a complete re-run of the pebble game otherwise.

Additional benchmarking runs were attempted for proteins with more than 80,000 atoms. However, the initial step of loading the PDB data via BioPandas [49] required an exponential amount of RAM (more than 200 GB), far exceeding the limits of normal research computers and even compute servers. This highlights an important limitation to our approach. Notably for trajectory analysis, the frame-based parallelization of both the detection of non-covalent interactions and the stability analysis with the pebble game allowed for an around 13 times faster overall calculation.

**Fig. 6:**
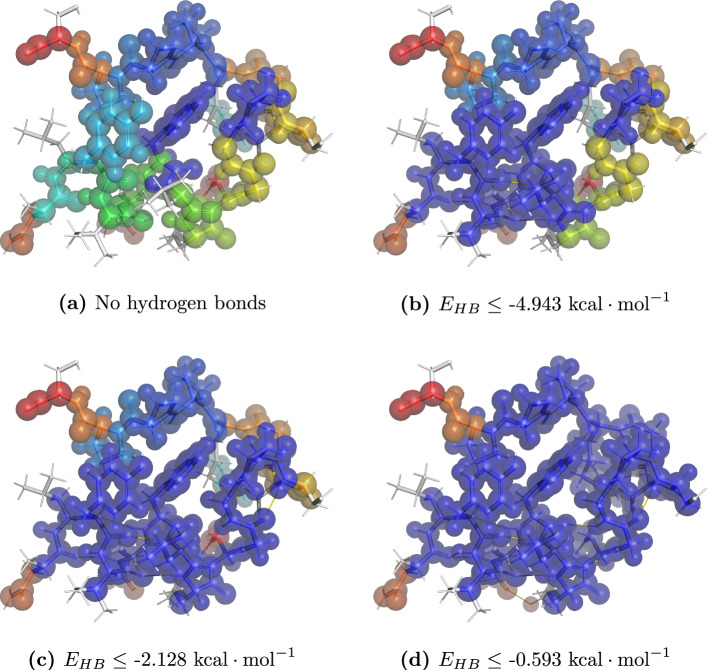
Result visualization for the dilution analysis of Trp-Cage Miniprotein Construct TC5b (PDB-code 1L2Y) at four selected energy thresholds for included hydrogen bonds ($E_{HB}$). Identical colored (connected) atoms indicate membership towards the same rigid region. Images were generated using PyMol [50]

As mentioned earlier, TRAMbio currently relies on the 3D molecular visualizer PyMol [50] for external visualization of protein analysis results. As an example, the visualization of four separate dilution steps of the *de-novo* protein Trp-Cage Miniprotein Construct TC5b [55] can be seen in Fig. 6.

Trivial components, i.e., every atom with only its direct covalent neighborhood, are not visualized in order to better highlight the remaining, more complex components. Without any hydrogen bonds, the protein is decomposed into a number of small rigid units (cf. Fig. 6a). Introducing more hydrogen bonds increases the overall rigidity until the majority of the protein forms a single rigid body (cf. Fig. 6d). The remaining non-trivial rigid structures mainly correspond to peripheral peptide units.

**Fig. 8:**
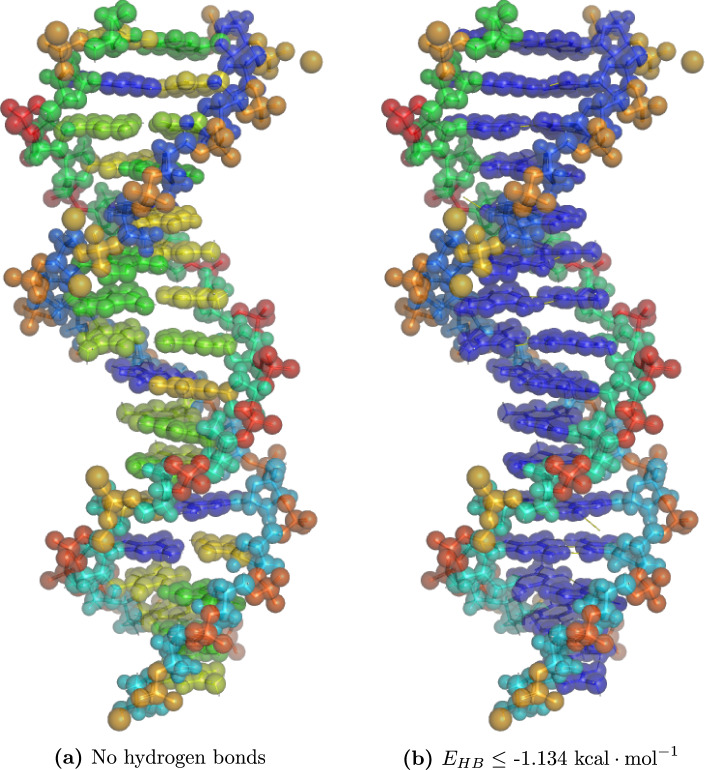
Application of dilution analysis workflow to a 16-base-pair B-DNA (PDB-code 3BSE). Panel (**a**) shows the molecule with no hydrogen bonds placed. As expected, the phosphate and ribose units along the backbone are rigid themselves but connected by still rotateable bonds. Each nucleobase also forms a rigid unit itself. After the placement of all 39 detected hydrogen bonds with bond energies $E_{HB}\le -1.134\, \text {kcal}\cdot \text {mol}^{-1}$, Panel (**b**) shows that each base pair has become rigid. Placement of missing hydrogen atoms was calculated using Reduce [53]. Images were generated using PyMol [50]

Our main focus was on the utilization of the pebble game for protein analysis. However, the application of the pebble game and in turn of TRAMbio is not limited to just proteins and the analysis workflows can be applied onto various different molecules, most notably DNA and RNA. To ensure this compatibility, the validation experiments run by the automatic test pipeline^8^ include the detection of characteristic hydrogen bonds between DNA or RNA nucleotides as well as the detection of $\pi$-stacking interactions in RNA structures. An exemplary application of rigidity analysis onto DNA, specifically a 16-base-pair B-DNA (PDB-code 3BSE) can be seen in Fig. 8.

**Fig. 9:**
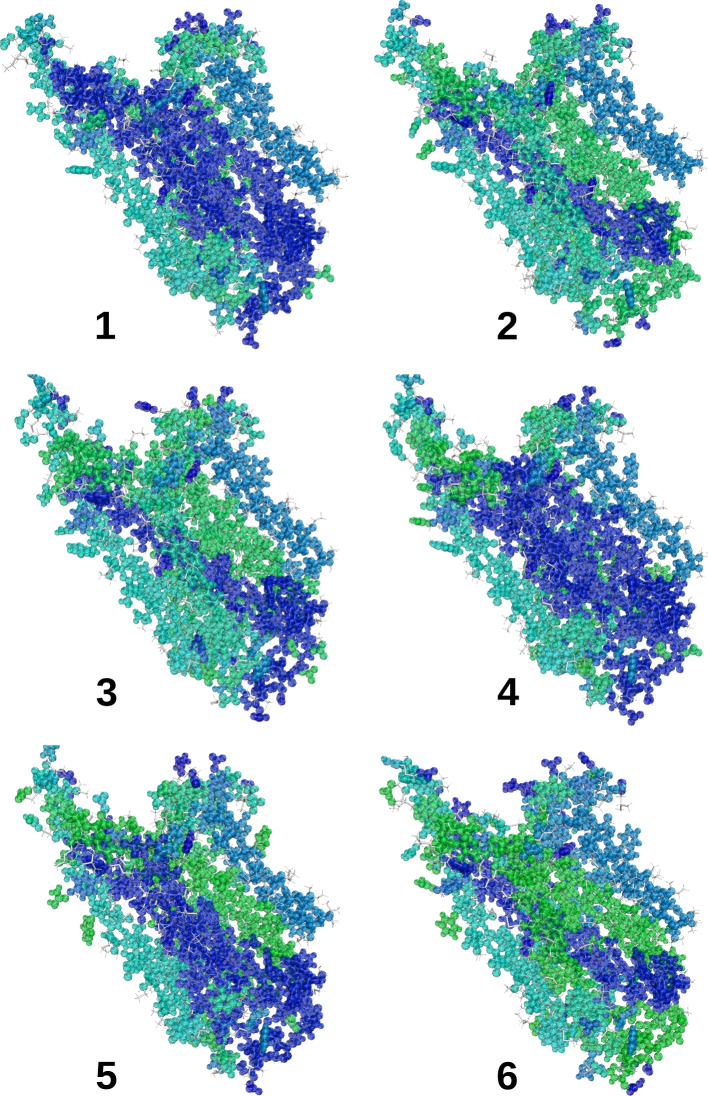
Application of rigidity-based community analysis to the $\beta$2-adrenergic receptor ($\beta$2AR), a large multidomain membrane protein with flexible regions. Panels 1–6 show the evolution of rigid amino acid communities across consecutive MD simulation frames, separated by 1 ns. Distinct colors denote individual communities, and their rearrangements highlight changes in rigidity patterns over time. This illustrates how the method can capture domain-level organization as well as the dynamic reorganization of flexible and disordered regions, providing insights into the structural basis of receptor stability and conformational transitions. Images were generated using PyMol [50]

To demonstrate the applicability of rigidity-based community analysis to large and complex proteins we use $\beta$2-adrenergic receptor ($\beta$2AR) as an example, see Fig. 9. The multidomain G protein-coupled receptor $\beta$2AR contains both rigid transmembrane regions and flexible loops. Figure 9 illustrates the evolution of rigid amino acid communities over six consecutive frames of a MD simulation, sampled at 1 ns intervals. Each community is represented by a distinct color, allowing dynamic changes in the underlying rigidity pattern to be visualized directly. The results reveal clear temporal reorganization of communities, with merging and splitting events occurring across transmembrane helices and intracellular loops. Such changes reflect local adaptations in structural rigidity and flexibility that are not easily captured by conventional secondary-structure or root-mean-square deviation (RMSD) analyses. Importantly, rigidity-based community analysis is able to highlight the interplay between relatively stable transmembrane domains and more disordered regions, thereby providing a domain-level perspective on structural stability while also capturing transient reconfigurations. This example demonstrates how rigidity-based community analysis can extend beyond small test proteins to large, multidomain membrane receptors. By tracking changes in community organization over time, the approach provides mechanistic insights into conformational transitions and dynamic coupling between structural regions, which are central to receptor activation and signal transduction.

## Concluding remarks

TRAMbio is a (pure-)Python package implementing the pebble game for $(k,\ell )$-sparse graphs and wrapper functions geared towards the straight forward analysis of general graphs and protein rigidity. Due to the lack of other accessible software, TRAMbio’s open-source framework bridges the gap in currently available rigidity software surrounding the pebble game. Our tool will therefore aid researchers in a wide spectrum of applications relating to protein stability and motion, such as characterizing motion mechanisms in proteins [56, 57] or simulated unfolding [11]. Additionally, as demonstrated in [38], graph rigidity results can be used for pre-processing in molecular dynamics. Since these faster heuristics can identify fully rigid atom communities as well as motion potential between them, rigidity results allow for a more compact protein modeling, in turn reducing the cost of more complex, geometric-based protein simulations. Our benchmarks highlight relatively low computational costs, even for very large proteins and time series. Already implemented parallelization can be used to further speed up computations. Deeper parallelization strategies may be implemented in a future update, but require further research. Although dilution analysis slows down computation times for proteins considerably, they are essential to determine correct energy thresholds, thereby avoiding even more costly reruns.

Future developments of TRAMbio will focus in particular on a custom graphical user-interface (GUI) for the protein analysis with a directly integrated molecular visualizer for rigidity results. This would simplify various use cases, such as the fine-tuning of parameters for dilution experiments. Another task in focus is additional support for analyzing allosteric effects [9] and the detection of symmetries in molecular graphs, which may lead to increased flexibility [58]. This is of interest both for small molecules and for protein complexes. The integration of a programming interface for defining custom interactions and energy functions would allow researchers to better fine-tune the mechanical modeling to the specific needs of their research task.

Editorial Policies for:

Springer journals and proceedings: https://www.springer.com/gp/editorial-policies

Nature Portfolio journals: https://www.nature.com/nature-research/editorial-policies

*Scientific Reports*: https://www.nature.com/srep/journal-policies/editorial-policies

BMC journals: https://www.biomedcentral.com/getpublished/editorial-policies

## Figures and Tables

**Fig. 7:**
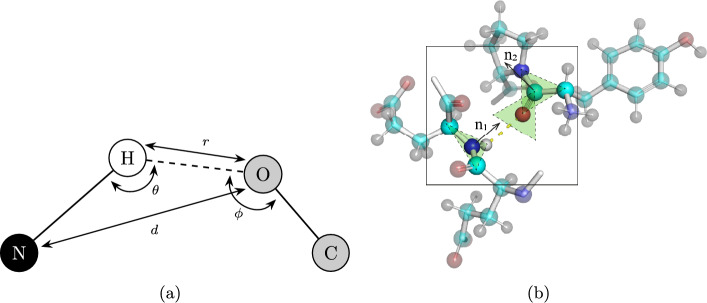
Geometric terms used in calculating hydrogen bonding energy potential (**a**). The donor-acceptor-distance *d* and hydrogen-acceptor-distance *r* for hydrogen bonds have cutoffs depending of the atoms involved. These are (i) the regular case with $d\le 3.6\, {\stackrel{\circ}{\text{A}}}$ and $r\le 2.8\, {\stackrel{\circ}{\text{A}}}$, (ii) the sulfur case (having one or more sulfur atoms are involved) with $d\le 4.2\, {\stackrel{\circ}{\text{A}}}$ and $r\le 3.0\, {\stackrel{\circ}{\text{A}}}$, in accordance with the expected lengths in hydrogen-bonded Dicysteine of approx. $4.15\, {\stackrel{\circ}{\text{A}}}\text { and }2.8\, {\stackrel{\circ}{\text{A}}}$ respectively [62], and (iii) the salt-bridge case (having charged acceptor and donor atoms) with $d\le 4.6\, {\stackrel{\circ}{\text{A}}}$ and $r\le 3.6\, {\stackrel{\circ}{\text{A}}}$. Additionally, *d* is required to be greater or equal to $2.6\, {\stackrel{\circ}{\text{A}}}$ to preliminarily exclude covalent distances. The angular terms are the donor-hydrogen-acceptor angle $\theta$ (required to be $\ge 80{^{\circ }}$), the hydrogen-acceptor-base angle $\phi$, and, if both donor and acceptor atoms are sp$^2$-hybridized, the angle $\gamma$ (not directly shown), which is between the normal vectors $n_1$ and $n_2$ of the planes of both sp$^2$-centers (**b**). As an oxygen’s sp$^2$-center cannot be directly calculated, the normal vector of the coinciding plane of the base atom is used instead. If $\gamma$ is below 90${^{\circ }}$, the supplement of the angle is substituted. Figure (**a**) adapted from [11]

## Data Availability

Benchmark protein datasets were taken from RCSB PDB. All software is available via Git: https://github.com/gate-tec/TRAMbio

## Appendix A: Graph construction

The following paragraphs elaborate further on the construction process for molecular graphs in TRAMbio.

***Covalent edges***

Similar to KINARI [21], the majority of covalent bonds is placed via a template for each residue, since the structure of amino acids and nucleotides is known a priori. These templates assume standard/non-degenerate amino acids or nucleotides with the main-chain modeled as a straight, directed graph from the nitrogen-terminus (N) to the carbon-terminus (C) for amino acids, where the main-chain oxygen (O) is separately attached to, and from the backbone phosphor atom (P) to the 3’-oxygen (O3’) for nucleotides. Ring structures, like the ribose sugar, are broken up at a pre-set bond and later re-connected in the pebble game. The side chains are modeled as a directed tree towards the root at the respective main-chain alpha carbon (CA) or 1’-carbon (C1’). Atom names in amino acids, peptides and nucleotides are specified using the IUPAC^9^ and IUBMB^10^ nomenclature [59].

Peptide bonds between two amino acids can be recognized from the sequence distance in the PDB data (provided that both amino acids lie on the same chain). Potential bonds are then double checked whether the covalent distance between the respective main-chain nitrogen (N) and carbon (C) falls in the range of peptide bonds. The calculation of phosphodiester bonds between DNA- or RNA-residues is analogous, using the backbone phosphor atom (P) and the 3’-oxygen (O3’). Checks are performed using the standard covalent radii specified via the atom classes in [60] and [61], which are also used for identification of covalent bonds in unrecognized structures like protein caps or ligands. Disulphide bonds are identified in the same way using the expected distance from [62].

***Hydrogen bonds & salt-bridges***

The approach for calculating hydrogen bonds is similar to the one in MSU ProFlex^11^ or ASU FIRST [10, 11]. A collection of potential bonds is defined via a preliminary distance cutoff for all pairs of valid donor and acceptor atoms. In addition to the residue-specific properties, simplistically, every nitrogen with bonded hydrogen atom is considered a valid hydrogen donor. This especially includes main-chain nitrogen (except for Proline) and every protonated amino group (-NH $_3^+$), the latter also being considered a suitable cation atom. In the same regard, every main-chain oxygen (O) or terminal oxygen (OXT) is considered an acceptor for a maximum of two hydrogen bonds. For a residue with a carboxyl terminus, both O and OXT are additionally considered suitable anionic atoms. The same follows suite for OP1 and OP2 bonded to the backbone phosphor atom in a DNA or RNA residue. This characterization allows for the handling of salt bridges as a special case of hydrogen bonds, if a cation and an anionic atom are within the constraints of the respective geometric criteria [11]. Furthermore, similar to MSU ProFlex, an additional case was included for hydrogen bonds involving any sulfur atoms, that has slightly relaxed criteria.

The geometric parameters used can be seen in Fig. 7. The strength evaluation of the hydrogen bonds and salt bridges respectively uses a modified Mayo-potential [51], which is based on the Lennard–Jones potential with corresponding values from the DREIDING molecular model [63]. The formulas used in TRAMbio are identical to the ones stated by [11].

In detail, the hydrogen bond potential is

A1 $$\begin{aligned} E_{HB} = V_0\cdot \left[ 5\cdot \left( \frac{R_0}{d}\right) ^{12} - 6\cdot \left( \frac{R_0}{d}\right) ^{10} \right] \cdot F(\theta , \phi , \gamma ) \end{aligned}$$

with constants $V_0=8\, \text {kcal}\cdot \text {mol}^{-1}$ and $R_0=2.80\, {\stackrel{\circ}{\text{A}}}$, being the well-depth and equilibrium distance respectively, and the salt-bridge potential is

A2 $$\begin{aligned} E_{SB} = V_S\cdot \left[ 5\cdot \left( \frac{R_S}{d+a}\right) ^{12} - 6\cdot \left( \frac{R_S}{d+a}\right) ^{10} \right] \end{aligned}$$

with constants $V_S=10\, \text {kcal}\cdot \text {mol}^{-1}$, $R_S=3.20\, {\stackrel{\circ}{\text{A}}}$ and $a=0.375\, {\stackrel{\circ}{\text{A}}}$. The angular term in Equation A1 depends on the hybridization states of both donor and acceptor atoms and infers the favorability of their three-dimensional orientation. This is due to the orbital hybridization influencing an atoms bonding properties [64]. The exact formulas are

A3 $$\begin{aligned} F=\left\{ \begin{array}{ll} \cos ^2 \theta \, \textrm{e}^{-(\pi - \theta )^6} \cos ^2(\phi - 109.5{{^{\circ }}}) & \text {if donor sp}{^3,} \text { acceptor sp}{^3}\\ \cos ^2 \theta \, \textrm{e}^{-(\pi - \theta )^6} \cos ^2 \phi & \text {if donor sp}{^3},\text { acceptor sp}{^2}\\ \cos ^4 \theta \, \textrm{e}^{-2(\pi - \theta )^6} & \text {if donor sp}{^2}, \text { acceptor sp}{^3}\\ \cos ^2 \theta \, \textrm{e}^{-(\pi - \theta )^6} \cos ^2(\max (\phi , \gamma )) & \text {if donor sp}{^2}, \text { acceptor sp}{^2} \end{array}\right. \end{aligned}$$

with angles in radians, if not indicated otherwise. If both donor and acceptor atoms are sp$^2$-hybridized, the angular term also takes into account, the attractive or repulsive force between the two respective sp$^2$-centers. This is factored in using the angle $\gamma$ between the normals of the two planes defined by the sp$^2$-centers (cf. Fig. 7b).

In addition to a cutoff value for hydrogen bonds at $-0.1\, \text {kcal}\cdot \text {mol}^{-1}$ by default (identical to [11]), an optional parameter for modeling weak hydrogen bonds through a gradually fewer number of bars was added, as showcased for KINARI [40].

***Hydrophobic interactions***

The hydrophobic effect describes the tendency of non-polar regions of molecules to aggregate near each other, thereby reducing the molecule’s surface exposed to water. It is usually quantified by the difference in free energy of water surrounding a molecule [65]. Regarding the pebble game, however, atomic interactions can only be modeled through bars limiting a certain number of degrees of freedom. TRAMbio features two ways of calculating hydrophobic interactions, which can be switched between and customized via environment variables.

The first approach is purely distance based, i.e., a hydrophobic interaction is placed for every carbon–carbon, carbon–sulfur, or sulfur–sulfur pair, when their van der Waals surfaces are within a cutoff distance of $0.25\, {\stackrel{\circ}{\text{A}}}$ [11]. The values for the van der Waals radii were taken from [66].

The second approach, originally proposed for KINARI [40], uses a Lennard–Jones-12-6 potential with the atom-dependent force field parameters taken from https://github.com/openforcefield/smirnoff99Frosst/blob/master/smirnoff99frosst/offxml/smirnoff99Frosst-1.1.0.offxml#L305. Since the second approach determines a quatified bond strength, the resulting hydrophobic interactions are also considered in the dilution analysis.

***Other interactions***

In addition to hydrogen bonds and hydrophobic interactions, $\pi$- and T-stacking as well as Cation-$\pi$ interactions belong to the set of calculated non-covalent bonds in TRAMbio’s modeling process. Their geometric parameters were taken from https://getcontacts.github.io/interactions.html. However, the list of aromatic rings was extended to account for DNA and RNA residue and the characterization of cations is identical to the one mentioned for salt-bridges above.

Atomic interactions specified in the PDB data, e.g. from LINK and/or CONECT entries, are by default included in the graph construction. This behavior can as well be toggled via an environment variable.

## Appendix B: Examples

See the Figs. 8 and 9.

## Footnotes

1 Except in proline residues.

2 http://graphml.graphdrawing.org/.

3 By providing a four-letter PDB-ID instead of an input file path.

4 Despite having the same time complexity.

5 Since the resources for ASU FIRST at http://flexweb.asu.edu/ and KINARI at http://kinari.cs.umass.edu are unavailable as of date.

6 All edges being directed towards the root.

7 Since the PDB data was not downloaded using TRAMbio itself only local loading times are included in benchmarks.

8 https://github.com/gate-tec/TRAMbio.
